# Circulating Level of CTRP1 in Patients with Nonalcoholic Fatty Liver Disease (NAFLD): Is It through Insulin Resistance?

**DOI:** 10.1371/journal.pone.0118650

**Published:** 2015-03-13

**Authors:** Parisa Shabani, H Naeimi Khaledi, Maani Beigy, Solaleh Emamgholipour, Eskandar Parvaz, Hossein Poustchi, Mahmood Doosti

**Affiliations:** 1 Department of Biochemistry, Faculty of Medicine, Tehran University of Medical Sciences, Tehran, Iran; 2 Students’ Scientific Research Center, Tehran University of Medical Sciences, Tehran, Iran; 3 Department of Clinical Biochemistry, Faculty of Medical Sciences, Tarbiat Modares University, Tehran, Iran; 4 Liver and Pancreatobiliary Diseases Research Center, Digestive Diseases Research Institute, Tehran University of Medical Sciences, Tehran, Iran; University College London, UNITED KINGDOM

## Abstract

Nonalcoholic fatty liver disease (NAFLD) is considered as one of the most common liver diseases. It is robustly linked to obesity and insulin resistance and is regarded as hepatic manifestation of metabolic syndrome (MetS). Adipokines are involved in the pathophysiology of liver diseases. The aim of this study was to evaluate the plasma concentrations of CTRP1 (complement-C1q TNF-related protein 1) in 22 patients with NAFLD, 22 patients with type 2 diabetes mellitus (T2DM), 22 patients with NAFLD+T2DM and 21 healthy controls, as well as their correlation with the level of metabolic and hepatic parameters. Plasma concentration of CTRP1 was measured with ELISA method. Plasma concentration of CTRP1 in patients with NAFLD, T2DM and NAFLD+T2DM were significantly higher than healthy subjects (*p*<0.0001). Moreover, we observed significant positive correlations between plasma level of CTRP1 and fasting blood glucose (FBG) (*p*<0.001), homeostasis model assessment of insulin resistance (HOMA-IR) (*p*<0.001), body mass index (BMI) (*p* = 0.001), alanine amino transferase (ALT) (*p* = 0.002), gamma glutamyl transferase (γ-GT) (*p*<0.001) and liver stiffness (LS) (*p*<0.001). Our results indicate the strong association of CTRP1 with insulin resistance in NAFLD. Also, it seems that CTRP1 can be considered as an emerging biomarker for NAFLD, however, more studies are necessary to unravel the role of CTRP1 in NAFLD pathogenesis.

## Introduction

Nonalcoholic fatty liver disease (NAFLD) is the most common liver disease which has a progressive nature and manifests with a wide spectrum ranging from lipid accumulation in hepatocytes (steatosis) to nonalcoholic steatohepatitis (NASH) which could progress to cirrhosis and hepatocellular carcinoma at the severe end of the spectrum [1–6].

Many lines of evidence indicate that NAFLD is associated with obesity, insulin resistance and metabolic syndrome (MetS). In fact, fatty liver is believed to be hepatic manifestation of MetS [7,8]. Regarding the role of adipokines in these complications, several studies have been performed to determine association of adipokines with NAFLD. Some studies have suggested that circulating levels of adipokines could be exploited as an additional tool for NAFLD detection [9–11].

CTRP1 (complement-C1q TNF-related protein 1) is a member of CTRP superfamily which comprises over thirty secreted proteins. They are called CTRPs since all members of the superfamily containing adiponectin share a c-terminal C1q globular domain which has a 3D structure similar to tumor necrosis factor alpha (TNF-α) [12–14]. CTRP1 is expressed in a wide range of tissues including heart, placenta, liver, muscle, kidney, prostate, and ovary but the highest expression level occurs in adipose tissue [14–18]. A more recent study has suggested possible role of CTRP1 in protection against insulin resistance and glucose intolerance of rats when challenged with high fat diet [13]. Administration of rosiglitazone has been turned out to regulate CTRP1 mRNA expression in adipose tissue of rosiglitazone-treated mice, which had been already demonstrated for adiponectin too. Furthermore, circulating level of CTRP1 has been indicated to increase in adiponectin-deficient mice. These studies have been provided encouraging evidence that CTRP1 might have a compensatory role in the loss of adiponectin [13,19].

Circulating level of CTRP1 has been evaluated in type 2 diabetes mellitus (T2DM), MetS and coronary artery disease (CAD) recently. Due to significant increment in serum level of CTRP1 in T2DM, it has been inferred that CTRP1 is involved in the pathogenesis of T2DM [15,20,21]. Regarding close association between diabetes and NAFLD, it is of interest to study the possible role of CTRP1 in NAFLD pathogenesis.

Thus far, no study has evaluated the plausible role of CTRP1 in NAFLD patients. Hence, the current study has been designed to measure the plasma concentration of CTRP1 in patients with NAFLD, T2DM and NAFLD with T2DM (NAFLD+T2DM) compared with healthy subjects and also to study its association with the metabolic and hepatic profile and also liver stiffness.

## Patients and Methods

### Participants

A total of 87 subjects including 22 with NAFLD, 22 with T2DM, 22 with NAFLD+T2DM and 21 healthy subjects participated in this study. The subjects were recruited from Shariati Hospital outpatient clinics from March 2012 until November 2013. Healthy subjects were also recruited from accompanying people. The study protocols were approved by Ethics Committee of Tehran University of Medical Sciences (TUMS) and informed consents were obtained from all subjects prior to study. Ultrasonography was used to diagnose NAFLD patients and liver stiffness was measured by a Transient Elastogeraphy (Fibroscan France). T2DM was diagnosed according to the basis of American Diabetes Association (ADA) criteria: a fasting blood glucose (FBG) ≥126 mg/dL or 2 hours blood glucose after a standard oral glucose tolerance test (OGTT) ≥200 mg/dL or random (non-fasting) blood glucose ≥ 200 mg/dL or HbA1c > 6.5%. Insulin resistance was determined by the modified homeostasis model assessment of insulin resistance (HOMA-IR) using the following formula: HOMA-IR = fasting blood sugar (mg/dL) × [fasting blood insulin (μU/mL)] / 405. All participants were men aged 43–72 years. In this study, subjects with excessive alcohol consumption (> 30 g/d) were not eligible. Participants were also excluded if they had severe concomitant disease including viral hepatitis (hepatitis B and hepatitis C), autoimmune liver disease, hemochromatosis, Wilson’s disease, renal disease and type 1 diabetes mellitus (T1DM). None of the patients were taking medication that has been reported to cause steatosis (steroids, estrogens, tamoxifen, amiodarone, valproic acid, diltiazem, or methotrexate). It should be noted that T1DM was diagnosed based on following criteria: diabetic ketoacidosis, acute ketonuria, age of onset, insulin deficiency and continuous treatment with insulin.

### Anthropometric and laboratory evaluation

Anthropometric indices of subjects including age, height, weight, blood pressure (BP), waist circumference (WC) were recorded. Body mass index (BMI) was calculated as body weight (kg) divided by the square of height (m^2^). WC was measured at the midpoint between the lowest rib and the iliac crest using a soft tape.

Blood samples were taken from all subjects after an overnight fasting. FBG was measured by commercially available kit based on the glucoseoxidase method. Insulin was assessed using enzyme linked Immunosorbent assay (ELISA) kit (Monobind Inc., USA) Serum total cholesterol (TC), triglycerides (TG), high-density lipoprotein cholesterol (HDL-C), low-density lipoprotein cholesterol (LDL-C), and levels of alanine amino transferase (ALT), aspartate amino transferase (AST), gamma glutamyl transferase (γ-GT), creatinin and urea were determined by enzymatic methods (Pars Azmoon kits, Iran) using full automated autoanalyzer.

### Liver stiffness (LS) measurement

LS was measured by transient elastography using the FibroScan 502 machine (EchoSense, Paris, France, 5MHz). According to the manufacturer’s guidelines the M probe was used for subjects with thoracic perimeter less than 110 cm and the XL probe for 110 cm and above. With the patient lying in the dorsal decubitus position with maximal abduction of the right arm, the probe was placed on the patient’s skin, overlying the right lobe of the liver, through the intercostal spaces. At least 10 measurements were done for each patient and the median value was recorded. Values were considered valid if the inter-quartile range (IQR) was less than 30% of the median reading.

### Measurement of plasma CTRP1

In order to measure CTRP1 of plasma, venous blood samples were drawn in EDTA-containing tubes. All samples were centrifuged for collection of plasma and stored at -80°C for subsequent analysis. CTRP1 levels were determined using a ELISA kit (Biovendor research and diagnostic products) with a minimum detectable concentration of 0.016 ng/ml, Intra assay Coefficients of Variability (CV) was 2.7% and Inter assay CV was 8.5%.

### Statistical analysis

Data are presented as means ± SEM and analysis was performed using SPSS 16 (SPSS, Chicago, IL, USA). Descriptive analysis was applied and all quantitative variables were tested for normality by means of the Shapiro-Wilk test prior to analysis. Comparisons between groups were carried out by Student’s t-test and analysis of variance (ANOVA), in case of normally distributed variables; and Mann-Whitney U and Kruskal-Wallis test for non-normally distributed variables. Tukey’s and Tamhane’s T2 post hoc analyses were employed after total significant ANOVA to discover the significant differences between group means, Bonferroni correction was performed in non-parametric post hoc analyses. Then, we performed analysis of covariance (ANCOVA) to remove the confounding effects. Multivariate linear regression (stepwise method) was utilized in order to identify the best regression model of CTRP1 levels. Also we conducted multinomial logistic regression to investigate the risk of diseases (NAFLD, T2DM, and NAFLD+T2DM) regarding CTRP1, anthropometric and metabolic features. Receiver Operating Characteristic (ROC) curve was also plotted using SPSS 16 to reflect the sensitivity and specificity of CTRP1 and other laboratory parameters in order to evaluate their ability to differentiate the investigated diseases. The comparison of the area under the curve (AUC) was performed by a p-value<0.05. The greater AUC represents the higher diagnostic value for CTRP1 and other parameters to differentiate the diseases.

## Results

Eighty seven men with the mean ± SEM age of 53.72 ± 0.81 years were recruited to our study. The clinical and laboratory characteristics of study participants are depicted in Table 1. Participants’ ages did not differ significantly among four groups (*p* = 0.366). Based on ANOVA; FBG (*p*<0.001), BMI (*p*<0.001), insulin (*p*<0.001), WC (*p*<0.001) and HOMA-IR (*p*<0.001) were significantly different among groups and post hoc analysis showed that the differences were mainly between healthy controls and the other three patient groups. Among patient groups, only 6 T2DM and 3 NAFLD+T2DM patients were under anti-diabetic medications. Additionally, 2 NAFLD, 6 T2DM, and 6 NAFLD+T2DM individuals were under antihypertensive medications.

**Table 1:** Anthropometric and laboratory characteristics of healthy subjects, NAFLD, T2DM and NAFLD+T2DM.

Characteristics		Healthy Subjects (N = 21)	NAFLD (N = 22)	T2DM (N = 22)	NAFLD+T2DM (N = 22)	Total Difference *p*-value
Age	years	53.95 ± 1.62	52.27 ± 1.38	55.95 ± 1.73	52.87 ± 1.70	0.366#
HOMA-IR	-	0.84 ± 0.12	2.63 ± 0.26	2.30 ± 0.28	3.02 ± 0.46	<0.001†
BMI	Kg/m^2^	24.76 ± 0.80	29.18 ± 0.50	27.28 ± 0.91	30.61± 0.84	<0.001†
FBG	mg/dL	89.68 ± 1.86	96.04 ± 1.56	154.11 ± 9.47	148.12 ± 12.77	<0.001#
Insulin	μU/mL	3.94 ± 0.51	11.05 ± 1.07	6.22 ± 0.76	8.79 ± 1.11	<0.001#
TG	mg/dL	121.13 ± 9.39	140.57 ± 8.16	139.21 ± 11.04	161.66 ± 20.18	0.218†
TC	mg/dL	190.01 ± 6.69	201.45 ± 7.64	195.84 ± 9.67	191.42 ± 16.17	0.831#
HDL-C	mg/dL	54.39 ± 2.78	48.61 ± 2.16	54.22 ± 2.88	49.79 ± 3.98	0.424†
LDL-C	mg/dL	111.65 ± 6.41	117.73 ± 7.75	113.76 ± 7.85	109.22 ± 10.53	0.903†
LDL-C/HDL-C	-	2.12 ± 0.14	2.42 ± 0.15	2.13 ± 0.13	2.04 ± 0.19	0.327†
TC/HDL-C	-	3.65 ± 0.16	4.24 ± 0.17	3.69 ± 0.14	3.59 ± 0.30	0.145#
Urea nitrogen	mg/dL	28.13 ± 1.17	32.29 ± 1.90	30.88 ± 1.33	30.98 ± 2.42	0.089#
Creatinin	mg/dL	1.27 ± 0.04	1.29 ± 0.04	1.25 ± 0.04	1.10 ± 0.08	0.312#
AST	U/L	17.58 ± 0.77	25.60 ± 2.54	17.20 ± 1.13	23.68 ± 2.46	<0.001#
ALT	U/L	15.87 ± 1.18	35.63 ± 4.57	18.09 ± 1.73	39.90 ± 4.41	<0.001#
γ-GT	U/L	21.20 ± 1.46	32.36 ± 2.81	30.25 ± 3.84	50.78 ± 9.01	0.001#
ALP	U/L	226.70 ± 10.52	237.41 ± 12.12	259.33 ± 19.77	210.57 ± 17.87	0.550#
LS	kPa	2.33 ± 0.48	5.46 ± 0.37	4.77 ± 0.32	7.00 ± 0.51	<0.001†
SBP	mmHg	127.65 ± 4.43	130.84 ± 4.49	136.79 ± 4.53	137.02 ± 4.40	0.372#
DBP	mmHg	78.15 ± 2.37	84.39 ± 3.76	79.95 ± 2.50	80.70 ± 2.10	0.708#
WC	cm	93.29 ± 2.14	104.95 ± 1.49	100.43 ± 2.38	109.61 ± 2.23	<0.001#

Data are expressed as means ± standard error of the means (SEM).

† One-way ANOVA;

# Kruskal-Wallis

**Fig 1:**
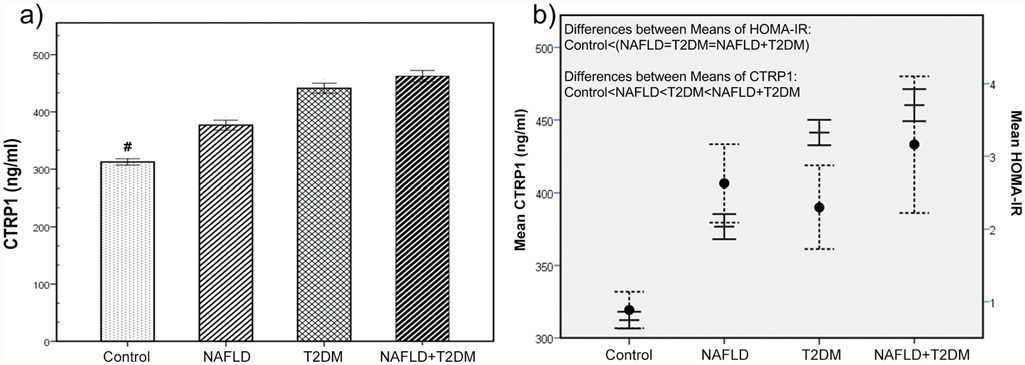
a) Graphic representation of the plasma levels of CTRP1 in NAFLD, T2DM and NAFLD+T2DM patients in comparison with control group. P-values were obtained by ANOVA and bars represent mean ± 95% confidence intervals. # represent p<0.0001. b) dual-axis representation for differences of CTRP1 and HOMA-IR between NAFLD, T2DM, NAFLD+T2DM, and healthy subjects. (Dashed lines represent HOMA-IR values while the non-dashed lines stand for CTRP1).

Fig. 1 describes the mean value of plasma CTRP1 and HOMA-IR among the study groups. Mean plasma concentration of CTRP1 is significantly different in the overall comparison of all four groups (*p*<0.0001). In comparison to control group, NAFLD group had higher plasma level of CTRP1 (*p*<0.0001), T2DM group showed higher plasma level of CTRP1 than NAFLD group (*p*<0.0001) and the difference (*p*<0.0001) in CTRP1 level between NAFLD+T2DM group and healthy controls was even greater compared to the two latter groups (fold change increase in NAFLD+T2DM = 1.48, fold change increase in NAFLD = 1.205; Fig. 1a). However, for HOMA-IR, only healthy subjects showed significant (*p*<0.001) lower levels than patients. No significant difference was observed between patients groups regarding HOMA-IR (Fig. 1b). Since there was a significant difference between healthy subjects and patients regarding HOMA-IR and BMI, we conducted ANCOVA to remove their effects from the observed difference of CTRP1 which showed us significant CTRP1 difference (p<0.0001) between groups even after removing HOMA-IR and BMI variance (S1 Table). Actually no significant differences were observed regarding the interaction of status (groups) × HOMA-IR or status × BMI. Furthermore, considering the close association between NAFLD and MetS, we categorized subjects into two groups: with and without MetS. CTRP1 was significantly (p<0.001) higher in MetS patients compared to healthy individuals. Thus, we carried out adjustment for possible effect of MetS on circulating level of CTRP1 in all studied groups (S2 Table). After removing the effects of MetS, the aforesaid differences between groups regarding CTRP1 were significant (p<0.001). We also performed adjustment for the effects of antihypertensive and anti-diabetic medications on levels of CTRP1 in four studied group separately (S3 Table). After adjusting for medications, significant differences remained in all four groups.

**Fig 2:**
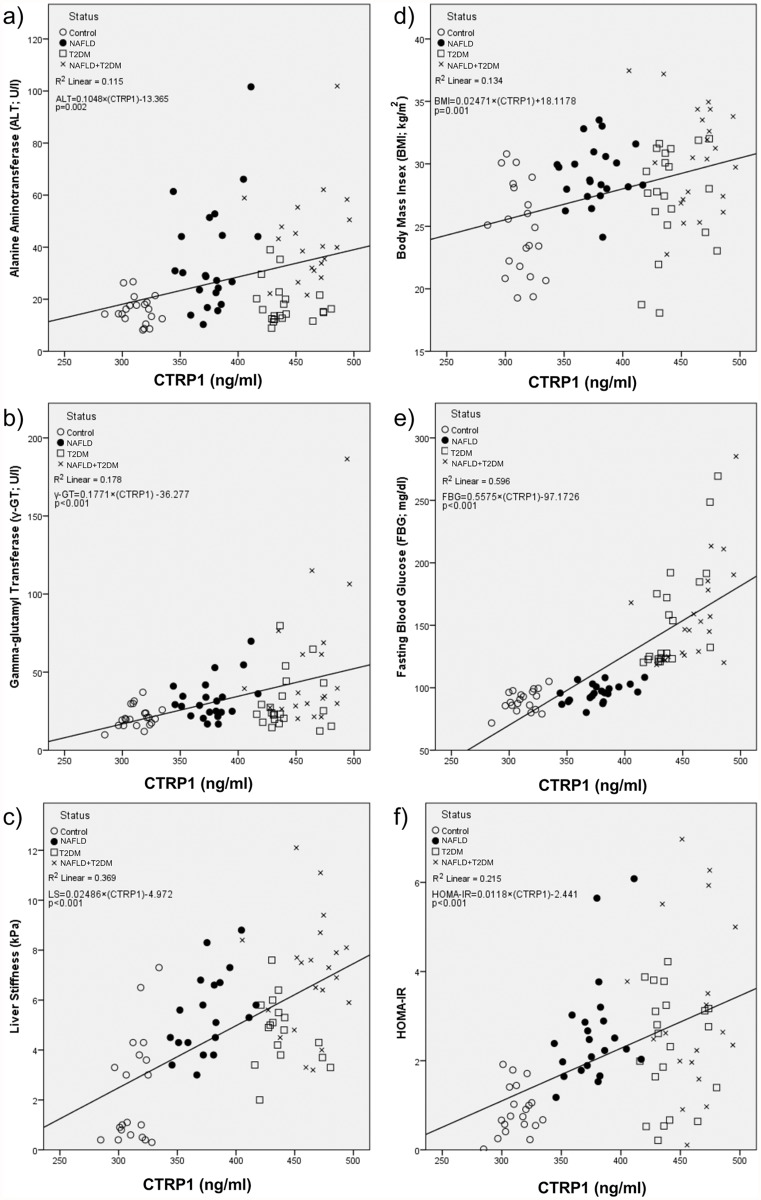
Significant positive correlations between plasma CTRP1 levels and serum ALT (a), γ-GT (b), LS (c), FBG (d), BMI (e), HOMA-IR (f) were found. Linear regression equation and r square (R^2^) are displayed at upper left position below the legends of participants’ status.

Also we assessed the correlation between plasma CTRP1 levels and liver enzymes, liver stiffness and metabolic profile in the whole study population. Plasma CTRP1 level showed a significant positive correlation with serum levels of liver enzymes including ALT and γ-GT (Fig. 2a and 2b). There was also significant positive correlation between plasma level of CTRP1 and liver stiffness (Fig. 2c); furthermore, significant positive correlations were found between plasma CTRP1 levels with BMI, FBG and HOMA-IR (Fig. 2d, 2e and 2f). Subgroup analyses revealed significant positive correlations between plasma level of CTRP1 and FBG in three patient groups, however correlations between plasma level of CTRP1 and liver stiffness was only observed in NAFLD group (Spearman Rho = 0.521; *p* = 0.011) and there was not a significant correlation between plasma level of CTRP1 and other parameters in subgroups.

Multinomial logistic regression (Table 2) was used to examine the associations between liver enzymes, liver stiffness and metabolic profile and the risk of all three conditions (NAFLD, T2DM, NAFLD +T2DM). Multinomial logistic regression analysis showed that prevalence odds for all three conditions increase (p<0.001) as plasma level of CTRP1 increases (Table 3a). However, after adjusting for HOMA-IR and BMI, although still remained significant (which is consistent with ANCOVA), odds ratios for all three conditions decreased markedly (Table 3c). Also the logistic regression model of interaction between CTRP1 and HOMA-IR and also CTRP1 and BMI were significant however with an almost negligible OR (Table 3d). Altogether these findings suggest that CTRP1 is highly associated with insulin resistance; however CTRP1 level is not confined to changes of HOMA-IR and it can also reflects liver stiffness.

**Table 2:** Multinomial logistic regression describes the association of CTRP1, clinical and metabolic characteristics and the outcome of NAFLD, T2DM and NAFLD+T2DM.

Characteristics	Outcome	NAFLD	T2DM	NAFLD+T2-DM
CTRP1	Odds Ratio (95% CI)	5.862 (4.868–7.061)	7.231 (6.997–7.472)	7.580 (7.580–7.580)
	*p*-value	<0.001	<0.001	<0.001
Age	Odds Ratio (95% CI)	.968 (.890–1.053)	1.034 (.956–1.119)	.980 (.903–1.063)
	*p*-value	NS	NS	NS
Insulin	Odds Ratio (95% CI)	1.715 (1.353–2.174)	1.276 (1.032–1.577)	1.557 (1.241–1.953)
	*p*-value	<0.001	0.024	<0.001
HOMA-IR	Odds Ratio (95% CI)	6.600 (2.589–16.826)	5.511 (2.183–13.911)	8.718 (3.353–22.666)
	*p*-value	<0.001	<0.001	<0.001
SBP	Odds Ratio (95% CI)	1.009 (.977–1.042)	1.023 (.992–1.056)	1.024 (.993–1.056)
	*p*-value	NS	NS	NS
DBP	Odds Ratio (95% CI)	1.041 (.988–1.098)	1.015 (.960–1.073)	1.021 (.967–1.077)
	*p*-value	NS	NS	NS
WC	Odds Ratio (95% CI)	1.132 (1.051–1.220)	1.074 (1.006–1.147)	1.199 (1.1031.303)-
	*p*-value	0.001	0.033	<0.001
BMI	Odds Ratio (95% CI)	1.391 (1.145–1.690)	1.193 (1.006–1.415)	1.587 (1.276–1.973)
	*p*-value	0.001	0.043	<0.001
FBG	Odds Ratio (95% CI)	1.113 (1.017–1.218)	8.737 (8.609–8.868)	8.779 (8.779–8.779)
	*p*-value	0.02	<0.001	<0.001
TG	Odds Ratio (95% CI)	1.009 (.995–1.022)	1.008 (.995–1.022)	1.018 (1.005–1.032)
	*p*-value	NS	NS	0.007
TC	Odds Ratio (95% CI)	1.008 (.992–1.024)	1.004 (.988–1.021)	1.012 (.996–1.029)
	*p*-value	NS	NS	NS
HDL-C	Odds Ratio (95% CI)	.954 (.901–1.009)	.99 (.949–1.051)	1.01(.951–1.053)
	*p*-value	NS	NS	NS
LDL-C	Odds Ratio (95% CI)	1.005 (.988–1.023)	1.002 (.984–1.020)	1.007 (.989–1.025)
	*p*-value	NS	NS	NS
Urea nitroen	Odds Ratio (95% CI)	1.098 (.999–1.207)	1.064 (.969–1.169)	1.137 (1.031–1.255)
	*p*-value	0.053	NS	0.01
Creatinin	Odds Ratio (95% CI)	1.743 (.073–41.716)	.628 (.024–16.138)	.192 (.007–5.428)
	*p*-value	NS	NS	NS
AST	Odds Ratio (95% CI)	1.218 (1.070–1.387)	.982 (.856–1.127)	1.222 (1.073–1.392)
	*p*-value	0.003	NS	0.003
ALT	Odds Ratio (95% CI)	1.221 (1.098–1.357)	1.057 (.956–1.169)	1.248 (1.121–1.390)
	*p*-value	<0.001	NS	<0.001
γ-GT	Odds Ratio (95% CI)	1.121 (1.032–1.218)	1.111 (1.023–1.208)	1.158 (1.065–1.260)
	*p*-value	0.007	0.013	0.001
ALP	Odds Ratio (95% CI)	1.003 (.993–1.014)	1.008 (.998–1.018)	1.001 (.991–1.012)
	*p*-value	NS	NS	NS
LS	Odds Ratio (95% CI)	2.607 (1.571–4.326)	2.107 (1.321–3.361)	3.917 (2.231–6.879)
	*p*-value	<0.001	0.002	<0.001

**Table 3:** Multinomial logistic regression for risk estimation of CTRP1 (a), HOMA-IR (b) and BMI (e) regarding the outcome of NAFLD, T2DM and NAFLD+T2DM.

Groups	B	SE	Wald	*p*-value	Odds Ratio	95% Confidence Intervalfor OR	Correct Prediction
a. Risk of outcomes along with each unit increase in CTRP1 (Multinomial Logistic Regression)							
NAFLD	1.769	0.095	347.469	<0.001	5.862	4.868–7.061	95.5%
T2DM	1.978	0.017	13996.452	<0.001	7.231	6.997–7.472	76.2%
NAFLD+T2DM	2.026	0.000	-	-	7.580	7.580–7.580	77.3%
b. Risk of outcomes along with each unit increase in HOMA-IR (Multinomial Logistic Regression)							
NAFLD	1.887	0.477	15.619	<0.001	6.600	2.589–16.826	45.5%
T2DM	1.707	0.472	13.052	<0.001	5.511	2.183–13.911	14.3%
NAFLD+T2DM	2.165	0.488	19.729	<0.001	8.718	3.353–22.666	40%
c. Adjustment of CTRP1 risk for HOMA-IR levels (Multinomial Logistic Regression)†							
NAFLD	1.100	0.093	138.890	<0.001	3.005	2.502–3.608	95.5%
T2DM	1.305	0.017	5630.394	<0.001	3.687	3.564–3.815	81%
NAFLD+T2DM	1.346	0.000	-	-	3.843	3.843–3.843	68.4%
d. Interaction risk (CTRP1×HOMA-IR) for each unit increase in both CTRP1 and HOMA-IR †							
NAFLD	0.006	0.002	14.495	<0.001	1.006	1.003–1.009	81.8%
T2DM	0.006	0.002	14.668	<0.001	1.006	1.003–1.009	0%
NAFLD+T2DM	0.007	0.002	19.068	<0.001	1.007	1.004–1.010	42.1%
e. Risk of outcomes along with each unit increase in BMI (Multinomial Logistic Regression)							
NAFLD	0.330	0.099	11.012	0.001	1.391	1.145–1.690	31.8
T2DM	0.176	.087	4.101	0.043	1.193	1.006–1.415	23.8
NAFLD+T2DM	0.462	0.111	17.235	0.000	1.587	1.276–1.973	65.2
f. Adjustment of CTRP1 risk for BMI (Multinomial Logistic Regression)†							
NAFLD	0.967	0.118	67.763	0.000	2.631	2.090–3.313	95.5
T2DM	1.201’	0.17	4727.439	0.000	3.323	3.212–3.429	76.2
NAFLD+T2DM	1.246	0.000			3.476	3.476–3.476	63.6
g. Interaction risk (CTRP1×BMI) for each unit increase in both CTRP1 and BMI							
NAFLD	0.002	0.001	11.132	0.001	1.002	1.001–1.003	63.6
T2DM	0.002	0.001	15.026	0.001	1.002	1.001–1.003	23.8
NAFLD+T2DM	0.003	0.002	23.021	0.000	1.003	1.002–1.004	63.6

Adjustment for risk of CTRP1 for the aforesaid outcome diseases by controlling for HOMA-IR (c) and BMI (f). The interactive risk of increasing HOMA-IR and CTRP1 levels (d) and also BMI and CTRP1 levels (g) for the aforesaid outcome diseases.

† Likelihood Ratio Test: *p*-value<0.0001

Having demonstrated the positive correlation between plasma CTRP1 levels and liver enzymes, liver stiffness and metabolic profile, we next performed stepwise linear regression analysis to investigate the significant determinants of CTRP1 concentration. It showed that in a model that explained 53.9% of circulating level of CTRP1; FBG and LS can predict (*p*<0.0001) CTRP1 concentration (linear equation [95% CI]: CTRP1 = 272.57 [241.44–303.7] + FBG × 0.62 [0.41–0.82] + LS × 10.691 [6.495–14.886]).

**Fig 3:**
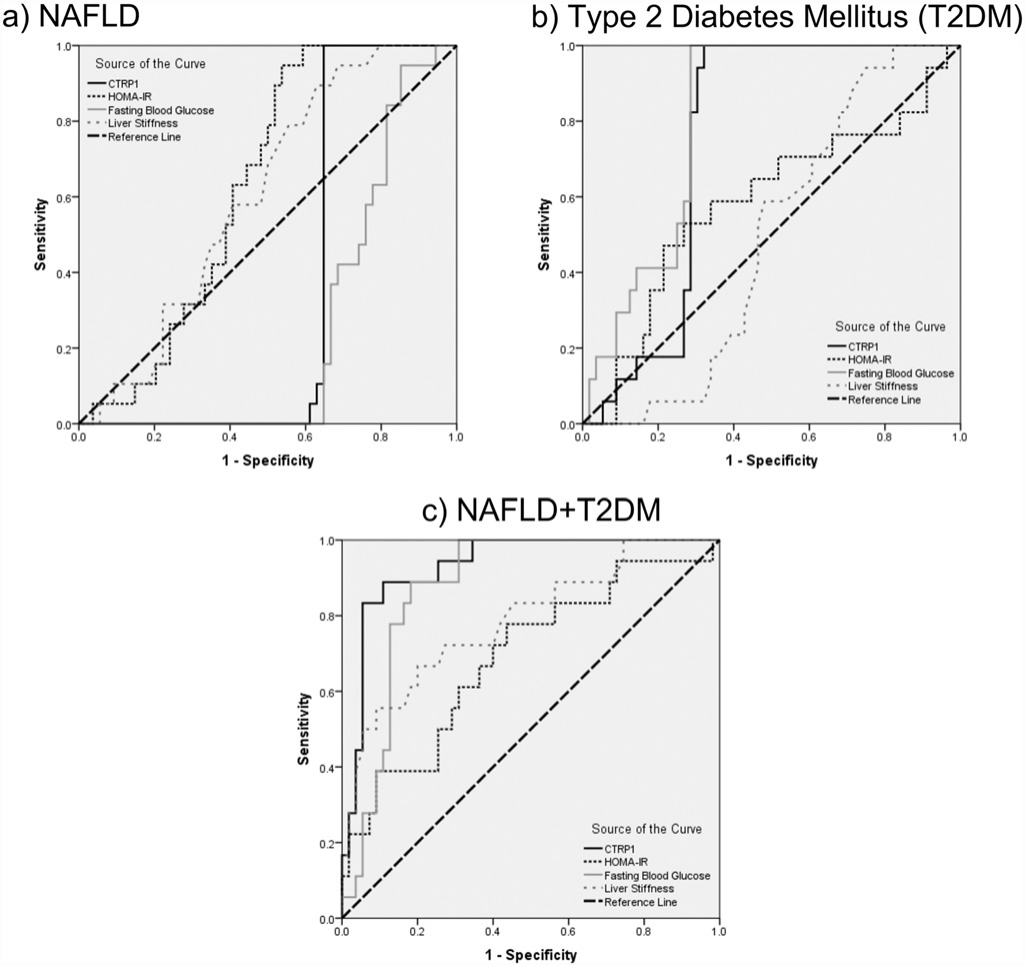
ROC curves for CTRP1, HOMA-IR, FBG, and liver stiffness are represented for a) NAFLD; b) T2DM; and c) NAFLD+T2DM patients.

The receiver operating characteristic (ROC) curves for NAFLD, T2DM, and NAFLD+T2DM regarding CTRP1, HOMA-IR, FBG, and LS are displayed in Fig. 3. Area under the curve (AUC) of CTRP1 (95% CI; *p*-value) was 0.355 (0.228–0.482; *p* = 0.061) for NAFLD (without T2DM) (Fig. 3a), 0.747 (0.637–0.857; *p* = 0.002) for T2DM (Fig. 3b), and 0.931 (0.871–0.991; *p*<0.001) for NAFLD +T2DM (Fig. 3c). CTRP1 did not provide a perfect sensitivity or specificity to differentiate NAFLD patients; thus we defined combinatory criteria to improve it. Actually, CTRP1≥350 ng/ml along with FBG<110 mg/dl could specifically differentiate NAFLD patients, with excellent diagnostic power (sensitivity = 90.91%; specificity = 100%; positive predictive value (PPV) for NAFLD without diabetes = 90.91%; negative present value (NPV) = 91.3%). But CTRP1 could not distinguish NAFLD from NAFLD+T2DM patients.

## Discussion

Compelling evidence has been provided revealing contribution of several factors including oxidative stress and inflammation in the pathogenesis of NAFLD, nevertheless, there is still a great deal of uncertainty in this regard [22–26]. Recent studies have paid particular attention to the role of adipokines in NAFLD pathogenesis [9–11]. Growing body of evidence points to the pivotal role of adipokines in insulin resistance which is a central factor in the development and even progression of NAFLD [27–32]. Among adipokines, CTRPs, as recently discovered family of adiponectin paralogs have been demonstrated to play important roles in energy homeostasis and insulin resistance [14–18]. However the contribution of CTRP1 in pathogenesis of NAFLD and its relation to insulin resistance have not been elucidated.

In the current study, we have found that plasma CTRP1 was significantly higher in the patient groups compared to healthy subjects, circulating level of CTRP1 was observed in increasing order in NAFLD, T2DM and NAFLD+T2DM respectively. Our findings regarding plasma concentration of CTRP1 and T2DM, were in accordance with the recent studies in which serum level of CTRP1 was significantly higher in T2DM, MetS and CAD patients compared to healthy people [15,20,21], however, in contrast to our results, another study demonstrated that circulating level of CTRP1 was significantly decreased in diet-induced obese mice. Overexpression of CTRP1 in transgenic mice led to insulin sensitivity improvement and decreased high-fat diet-induced weight gain. They attributed reduced adiposity to enhanced fatty acid oxidation and energy expenditure by AMP-activated protein kinase (AMPK) activation, which promotes mitochondrial fat oxidation in skeletal muscle [13]. Increased level of CTRP1 in our study can be interpreted as a compensatory mechanism for improving insulin resistance. In our study, we observed strong positive correlation between plasma level of CTRP1 and FBG and also HOMA-IR which was in consistent with previous reports in T2DM and MetS patients [15,20,33]. A growing body of evidence showed that hyperinsulinemia, as a hallmark of insulin resistance, led to de novo lipid synthesis and acceleration of lipid accumulation in liver. [13]. Hence CTRP1 might exert protective effect against fat deposition and subsequent liver injury via affecting insulin resistance. The fact that strong association between CTRP1 and NAFLD, nor is completely confounded by HOMA-IR neither completely interacted with it; supports the hypothesis of compensatory role of CTRP1 against insulin resistance and fatty liver.

Moreover, CTRP1 has been postulated to mediate some of the salutary metabolic effects of adiponectin [12,13]. Several studies have implicated that adiponectin-deficient mice showed no or mild difference in insulin resistance unless challenged with high fat diet [13,29,34]. Another study has demonstrated about 2-fold increase in circulating level of CTRP1 in adiponectin-null mice [18]. Based on these reports, they have suggested CTRP1 as a candidate which could compensate for the beneficial actions of adiponectin in the loss of adiponectin [12,13]. Put differently, the mentioned report of transgenic mice overexpressing CTRP1 has revealed significant decrease in serum level of adiponectin upon a metabolic challenge of high fat diet. Additionally, emerging evidence has revealed that circulating level of adiponectin was significantly lower in NAFLD patients compared to healthy people [35,36]. The present study demonstrated that circulating level of CTRP1 in both NAFLD and NAFLD+T2DM groups were significantly higher than control group which might confirm its plausible role as an adipokine accounting for metabolic actions of adiponectin in the loss of adiponectin.

In the current study, significant positive correlation was observed between circulating level of CTRP1 and markers of liver function and also liver fibrosis, liver enzymes (ALT and γ-GT) and liver stiffness respectively. Previous studies have indicated different level of adipokines such as leptin and adiponectin in fatty liver patients but they have not reported significant correlation between circulating adipokines and liver enzymes or liver stiffness [10,22,27].

One may inquire into whether or not CTRP1 could be considered as a diagnostic tool in NAFLD. Based on the AUC of ROC curve in NAFLD (without T2DM) patients it does not provide a perfect sensitivity or specificity; however after defining the criteria as CTRP1≥350 ng/ml along with FBG<110 mg/dl, the diagnostic power to differentiate NAFLD is considerably increased

Despite the advantageous findings of our study regarding the role of CTRP1 in NAFLD-related insulin resistance, we faced some limitations as we could not investigate other adipokines as the other possible pathogenic pathways involved with CTRP1 effects. It is highly desirable to conduct studies regarding CTRP1 role in pathogenesis of NAFLD with larger samples of NAFLD patients and also in prospective designs in NAFLD.

In conclusion, our study uncovered that changes of CTRP1 is associated with insulin resistance might be an emerging possible tool for NAFLD detection. However, more studies are needed to clarify the role of CTRP1 in the etiology of NAFLD.

## Supporting Information

S1 Table A full factorial model of ANCOVA in order to evaluate the effect of HOMA-IR and BMI as possible confounders on the plasma levels of CTRP1 in controls, NAFLD, T2DM and NAFLD+T2DM patients. (DOCX)

S2 Table A full factorial model of ANCOVA to adjust the effect of medication on the plasma levels of CTRP1 in controls, NAFLD, T2DM and NAFLD+T2DM patients. (DOCX)

S3 Table A full factorial model of ANCOVA to adjust the effect of MetS on the plasma levels of CTRP1 in controls, NAFLD, T2DM and NAFLD+T2DM patients. (DOCX)
