# Supplementary material for: Circulating Level of CTRP1 in Patients with Nonalcoholic Fatty Liver Disease (NAFLD): Is It through Insulin Resistance?
Source: PLoS One. 2015 Mar 13;10(3):e0118650. doi: 10.1371/journal.pone.0118650 (PMC4358971; doi:10.1371/journal.pone.0118650)
Supplement: S1 Table — (DOCX) [file pone.0118650.s001.docx]

Table S1: A full factorial model of ANCOVA in order to evaluate the effect of HOMA-IR and BMI as possible confounders on the plasma levels of CTRP1 in controls, NAFLD, T2DM and NAFLD+T2DM patients.

|  | |  | | | BMI and HOMA-IR matched | |  |
| --- | --- | --- | --- | --- | --- | --- | --- |
| (I) Status | (J) Status | Mean Difference (I-J) ± SEM | p-value | Mean Difference (I-J) ± SEM | | p-value | |
| Control | NAFLD | -64.13 ± 5.71 | <0.001 | -63.00 ± 6.93 | | <0.001 | |
|  | T2DM | -129.16 ± 5.71 | <0.001 | -128.09 ± 6.39 | | <0.001 | |
|  | NAFLD+T2DM | -150.45 ± 5.78 | <0.001 | -146.33 ± 7.95 | | <0.001 | |
